# Longitudinal trends of remodeling mechanisms after acute myocardial infarction based on severity of ischemic insult: A quantitative MRI study

**DOI:** 10.1186/1532-429X-13-S1-O57

**Published:** 2011-02-02

**Authors:** Nilesh R Ghugre, Jennifer Barry, Beiping Qiang, John J Graham, Kim Connelly, Alexander J Dick, Graham A Wright

**Affiliations:** 1 Sunnybrook Health Sciences Centre, Toronto, ON, Canada; 2 St. Michael's Hospital, Toronto, ON, Canada; 3 University of Ottawa Heart Institute, Ottawa, ON, Canada

## Introduction

In acute myocardial infarction (AMI), the aim of any therapeutic intervention is to reduce the infarct size and attenuate adverse remodeling. The type and extent of infarction encountered clinically [transmural, hemorrhagic, heterogeneous, with microvascular obstruction (MVO)], is primarily determined by the severity of the initial ischemic insult. Understanding the *in-vivo* pathophysiological mechanisms after AMI as a function of severity will be key in predicting functional recovery, prognosis and assessing the efficacy of novel therapies.

## Purpose

To evaluate longitudinal fluctuations in edema, hemorrhage and vasodilatory function in infarcted and remote territories of porcine myocardium following different ischemic insult durations.

## Methods

The study involved two groups of animals that were subjected to balloon occlusion of the LAD [90 min (N=4) and 45 min (N=3)], followed by reperfusion. Imaging was performed on a 3T MRI scanner (MR 750, GE Healthcare) pre-LAD occlusion, at day-2 as well as weeks- 1,2,4 and 6 post-LAD occlusion. Edema was evaluated by T2 quantification using a T2-prepared spiral sequence and hemorrhage was identified by T2* determined using a multi-echo gradient-echo acquisition. Vasodilatory function was assessed at rest and following Dipyridamole administration (stress), noting BOLD-induced T2 alterations. Non-infarcted basal myocardium was also analyzed to study remote zone remodeling. A contrast-enhanced IR-GRE sequence was used for infarct assessment.

## Results

**Figure 1:**
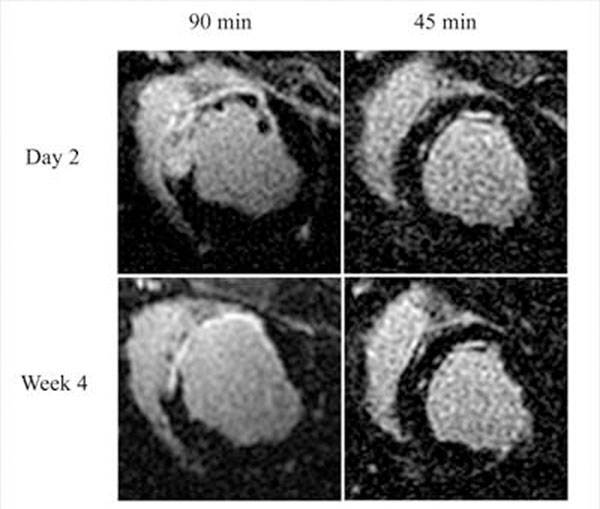
As demonstrated in these representative images, the 90min occlusion model consistently produced hemorrhagic transmural infarcts with MVO in all animals. In contract, infarction in the 45 min group was non-transmural, non-hemorragic and heterogenous.

**Figure 2:**
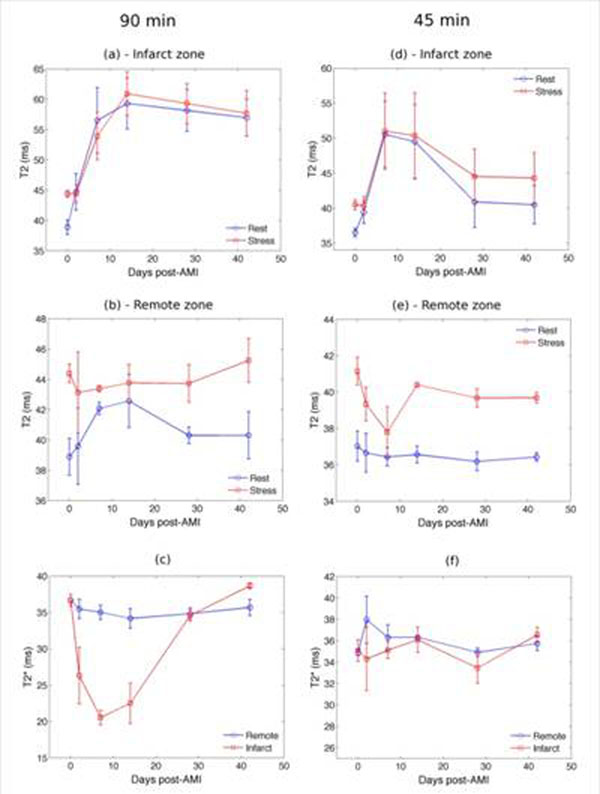
Cumulative time course of T2 and T2* parameters post-AMI pooled across all animals in the 90 (a-c) and 45 (d-f) min sub-groups: error bars show standard error and day 0 indicates control MRI. (a), (d) represent fluctuations in T2 within infarct zone while (b), (c) represent remote zone under rest and stress states. (c), (f) demonstrates T2* alterations in infarct and remote zones. At week 6, edema was apparent in the 90min group where as it was significantly reduced in the 45min group suggesting a quicker resolution of inflammatory response.

Figure 1 demonstrates contrast-enhanced short-axis slices from representative animals subjected to 90 and 45 min occlusion shown at day-2 and week-4 post-AMI. In the infarct zone, the 90 min group demonstrated significant elevation in resting T2 (Fig. 2a), persisting at week-6 (p<0.005) that was unchanged by stress. In the 45 min group, T2 values had normalized by week-6 (Fig. 2d) with non-significant elevation under stress. Vasodilatory function in the remote zone normalized after week-2 in the 45 min group (Fig. 2e) whereas it demonstrated prolonged impairment beyond week-2 in the 90 min animals (Fig. 2b). In the infarct zone of the 90 min group, T2* was significantly depressed between day-2 and week-2 (p<0.05) while it remained unaffected throughout infarct healing in the 45 min group.

## Conclusions

Quantitative *in-vivo* MRI evaluation of disease evolution can distinguish longitudinal trends of the underlying remodeling processes based on severity of the ischemic insult. MRI parameters revealed faster resolution of edema and earlier restoration of vasodilatory function in less severe infarcts potentially indicating reduced adverse remodeling. This characterization may allow evaluation of novel therapies targeted to alleviate ischemic injury and prevent MVO/hemorrhage.

